# Transition Between Diffuse Large B-Cell Lymphoma and Classical Hodgkin Lymphoma– Our Histopathological and Clinical Experience With Patients With Intermediate Lymphoma

**DOI:** 10.3389/pore.2021.625529

**Published:** 2021-03-30

**Authors:** Zsófia Simon, Bálint Virga, László Pinczés, Gábor Méhes, Zsófia Miltényi, Sándor Barna, Roxana Szabó, Árpád Illés

**Affiliations:** ^1^ Department of Hematology, Debrecen, Hungary; ^2^ Institutes of Pathology, Faculty of Medicine, University of Debrecen, Debrecen, Hungary; ^3^ Scanomed Kft, Budapest, Hungary

**Keywords:** gray zone lymphoma, hodgkin lymphoma, diffuse large B cell lymphoma, brentuximab vedotine, stem cell transplant

## Abstract

Even though information about the pathophysiology and clinical features of grey-zone lymphoma, an entity intermediate between classical Hodgkin lymphoma and diffuse large B-cell lymphoma, is growing, there are still a number of unanswered questions. The disease has no easily reproducible diagnostic criteria, which makes identification challenging. Uncommon, mixed histological picture and unusual clinical presentation should raise suspicion for grey-zone lymphoma. In this retrospective analysis we present 9 gray zone lymphoma patients, who were diagnosed in our institute between 2008 and 2018. The histological diagnoses was oftentime challenging, we asked for a revision in three cases due to the unusual clinical behavior and in other three cases only the relapse of the disease proved to be grey-zone lymphoma. Based on the initial histopathological diagnoses we applied adriablastine-bleomycine-vinblastine and procarbasine or cyclophosphamide-vincristine-adriablastine and prednisolon as first line chemotherapy regime with additional rituximab in six cases and brentuximab-vedotine in one patient. In six of the nine patients due to the primary refractory disease we used rituximab plus cisplatine, cytosine-arabinoside, prednisolone salvage treatment and five of these patients responded well enough to become eligible for autologous stem cell transplantation. One young male patient was refractory for various treatments and died due to the progression of his lymphoma. As a rare disease grey-zone lymphoma has no existing diagnostic criteria or guiedlines for its standard of care, which makes the everyday practice rather challenging for the clinicians, and emphasize the importance of unique decision making in every case and the repeated consultation between the pathologist and hematologist.

## Introduction

The morphological and immunohistochemical features of mediastinal gray zone lymphoma were first described by Traverse-Glehen in 2005 [1], to be later defined as a provisional independent entity in the WHO classification in 2008: B-cell lymphoma, unclassifiable, with features intermediate between diffuse large B-cell lymphoma (DLBCL) and classical Hodgkin lymphoma (cHL) [2]. Although there has been a considerable increase in the body of knowledge about the pathology and clinical features of the disease, there are still numerous unanswered questions. Due to its rarity its diagnosis presents a considerable challenge for even an experienced a hematopathologist. No consensus has yet emerged as to the defining diagnostic criteria, although, based on the results of retrospective studies, it can be concluded that typically, two histological patterns can be distinguished. One is a morphology corresponding to primary mediastinal large B-cell lymphoma (PMBL) or DLBCL accompanied by weaker or even absent CD20 (B-cell marker) expression and pronounced CD30 and/or CD15 expression. The other pattern shows cHL-type morphology as well as marked CD 20 positivity, and moderate CD30 expression [3–5].

Although the disease tended to be mediastinal in appearance in its first description, today it is clear that two, clinically different types can be distinguished; one with a predominantly mediastinal presentation (mediastinal gray zone lymphoma: MGZL), and the other with a disseminated presentation and with or without mediastinal involvement (non-mediastinal gray zone lymphoma: NMGZL). While the former is characterized by younger age, early stage, the presence of a bulky tumor, the latter tends to appear at an older age with bone marrow, and (multiplex) extranodal organ involvement [3].

There are no clear guidelines for the management of MGZL, either. Available literature suggests that, for first-line treatment, DLBCL protocols such as R-CHOP (rituximab, cyclophosphamid, adriablastin, vincristin, and prednisolone) are more effective than the gold standard the ABVD regime (adriablastin, bleomycin, vinblastin, and dacarbazine) used in cHL. Perhaps even more effective are dose-intensified protocols like DA-EPOCH (dose-adjusted etoposide, doxorubicin, cyclophosphamide, vincristine, and prednisolone) or escalated BEACOPP (bleomycin, etoposid, adriablastin, cyclophosphamid, vincristin, procarbazin, and prednisolone). At the same time, despite more intensive therapy, treatment outcomes lag behind those of treatments for PMBL and cHL [3–5]. Primary refractory and relapsed (early relapsed) diseases are also common, and can be treated rather effectively in eligible patients with a salvage protocol, followed by high-dose therapy and a consecutive autologous hematopoietic stem cell transplant (aHSCT) [4]. In spite of a more unfavourable clinical presentation, there are no significant differences between the survival rates of mediastinal and non-mediastinal forms [3]. Based on the characteristic/dominant cell surface antigen expression of malignant cells monoclonal antibody therapy/immunotherapy (anti-CD20, anti-CD79 or anti-CD30) has its role in the therapy of GZL, while the place of novel drugs (e.g. brentuximab-vedotin or PD1-inhibitors) in GZL management is not clear.

Despite a growing body of information it is clear that a practising clinician, in charge of treatment of a patient with GZL, faces numerous open questions and serious therapy decisions. Even though our knowledge of the literature is increasing, it is till rather scarce compared with other entities. All this has urged us to review our own patients’ data and summarize our experience.

## Patients and Methods

We reviewed the registries of patients with Hodgkin and diffuse large B-cell lymphoma at the Department of Hematology, University of Debrecen, looking for grey-zone lymphoma (GZL) cases that we had diagnosed and treated. Relying on the introduction of a lymphoma intermediate between cHL and DLBCL in the 2008 WHO classification we reviewed the period between January 1, 2008 and December 31, 2018, and found 9 patients whose clinical data and histopathological samples were available. GZL was suspected in further two cases but these had no histological blocks and their medical histories were incomplete, too, which led to their exclusion from the present study. In our study we included grey-zone lymphoma cases that were discovered during their first histological examination or a revision and also cases where GZL was diagnosed during a relapse. The study was retrospective in nature, thus no informed consent was required. We obtained our clinical data using our patient documentation system.

The histopathological examinations were performed at the Institute of Pathology, University of Debrecen in each case, involving processing and immunohistochemical staining of samples from lymph node excisional biopsy or core-needle biopsy. Immunostaining of formalin-fixed paraffin-embedded (FFPE) tissue specimens covered the examination of markers used in the diagnostics of Hodgkin and B-cell non-Hodgkin lymphomas (CD3, CD4, CD8, CD15, CD20, CD30, LCA, LMP1, Mib-1, MUM1, OCT-2, PAX-5, and PD-L1). Preparation of slides, antibody reactions and their detection were performed according to standard protocols, using the Leica BondMax automated system. Diagnosis of grey-zone lymphoma was based on a characteristic morphology and immunohistochemical features published in the WHO classification.

## Results

Of all the patients with B-cell lymphoma diagnosed and treated at the University of Debrecen during the period from January 1, 2008 to December 31, 2018 grey-zone lymphoma, an intermediate histological form between classic Hodgkin lymphoma and diffuse large B-cell was confirmed in 9 cases as primary diagnosis, with histological revision or during a relapse. In the given period we provided care for 257 newly diagnosed patients with Hodgkin lymphoma and 423 newly diagnosed patients with DLBCL. Patient characteristics are summarized in Table 1. The modest number of cases does not allow for statistical analysis or identification of significantly different characteristics.

**TABLE 1:** Clinical characteristics of nine grey-zone lymphoma patients.

Patient	Age (y)	Gender	Histological diagnoses	Stage	B-sign	Bulky	Extra nodal	1st line treatment	R	aHSCT	OS (mo)	Exit
#1	54	F	GZL	4	+	−	−	BV-CHOP	CMR	−	21	−
#2	74	F	GZL	4	+	−	−	R-CHOP	PR	−	22	+
#3	80	F	GZL	3	+	−	−	R-CHOP	PR	−	8	+
#4	17	M	cHL, revision: GZL	4	+	+	−	ABVD 1x R-CHOP	PR	+	100	−
#5	27	M	DLBCL, revision: GZL	2	+	+	−	R-CHOP-14	PR	+	138	−
#6	69	F	DLBCL, revision: GZL	4	−	−	+	R-CHOP	PR	+	20	−
#7	32	M	DLBCL rebiopsy due to progression: GZL	4	+	+	+	R-CHOP	PD	−	13	+
#8	54	F	cHL, relapse: GZL	2	+	−	−	−ABVD	CMR	+	117	+
#9	45	F	cHL, relapse: GZL	3	−	−	−	ABVD	CMR	+	106	−

R, response; aHSCT, autologous hematopoetic stem cell transplantation; OS, overall survival; mo, month; GZL, grey-zone lymphoma; cHL, classical Hodgkin lymphoma; DLBCL, diffuse large B-cell lymphoma; BV, brentuximab vedotine; CHOP, cyclophosphamid-adriablastin-vincristine-prednisolone; R, rituximab; ABVD, addriablastin-bleomycin-vinblastine-dacabazine; CMR, complete matabolic response; PR, partial response; PD, progressive disease.

Of the 9 patients 6 were females and 3 males, with a median age of 50.2 years (17–80 years) at the time of diagnosis. With the exception of two cases the disease was identified at an advanced stage with bone marrow involvement in 5 patients. We did not encounter any exclusively mediastinal localization; in one of the two early stage cases there was cervical involvement besides the mediastinum and in the other early stage case extensive supradiaphragmatic involvement was confirmed at the time of the diagnosis. In these cases mediastinal GZL, while in the other cases non-mediastinal GZL was surmised. B symptoms were typical, while occurrences of bulky tumor or extranodal involvement were less common.

**FIGURE 1:**
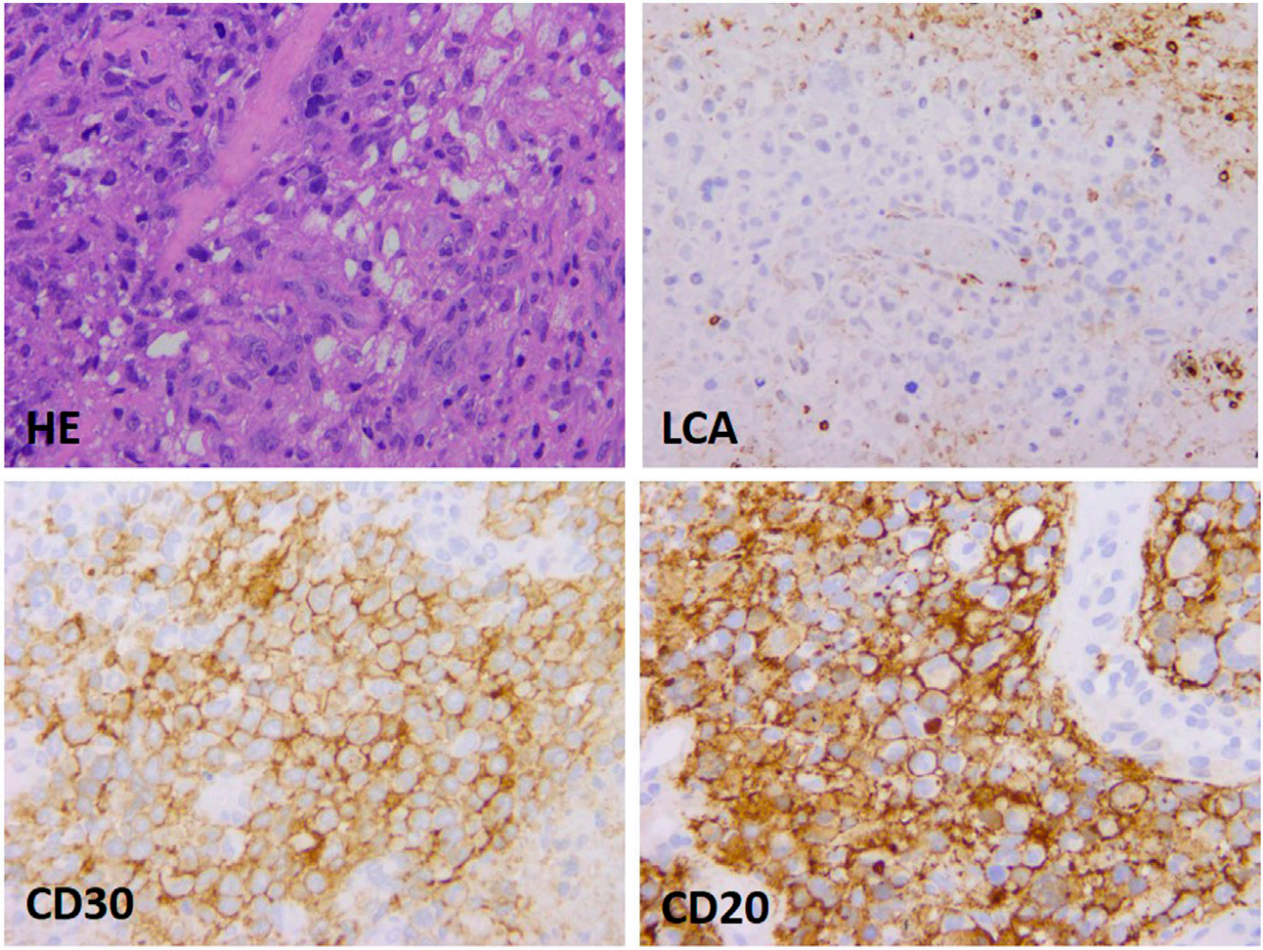
DLBCL-type presentation of mediastinal gray zone lymphoma. The histological picture shows moderate lymphoid infiltration in a relatively stroma-rich environment, the large atypical–blastoid cells are LCA negative, but characterized by intensive CD30 and CD20 expression (growthx40).

**FIGURE 2:**
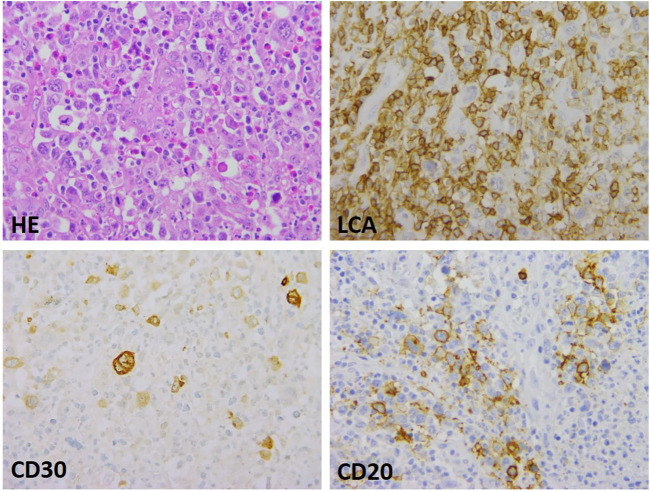
Mediastinal gray zone lymphoma with Hodgkin-type morphology. The intermediate nature manifests as atypical, HRS-type cell groups in a mixed inflammatory environment, as well as partial LCA, CD 30 and CD20 expression (growthx40).

The basis for a histological diagnosis was the presence of characteristic Hodgkin and Sternberg-Reed (HRS) cell morphology and CD30 and MUM1 (IRF4) immunoreactivity. In addition, distribution and immunophenotype of atypical cells were unique and varied. Morphologically two main histological patterns could be distinguished. One was characterized by DLBCL-type morphology with LCA, CD20, PAX5 and/or OCT-2 deficiency (6 cases); the other group included cHL/T-cell rich DLBCL-type cases with a rather high number of inflammatory and reactive immune cells as main characteristic features. Intensity of CD30 and CD20 positivity was also variable with the presence of mixed phenotype (3 cases). The histological pictures of the two patterns are shown in Figures 1, 2.

**FIGURE 3:**
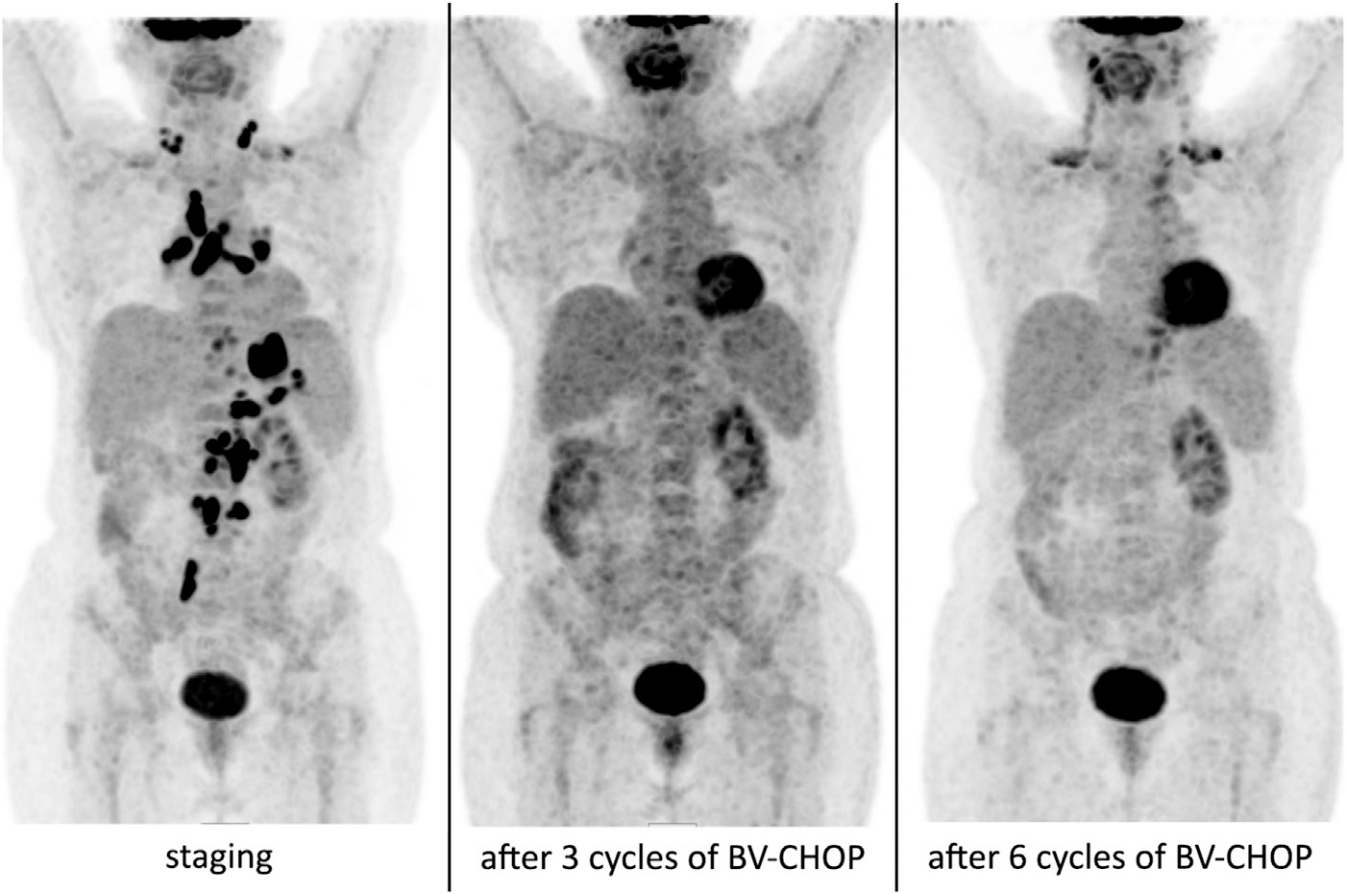
PET/CT scan results of our patient with grey-zone lymphoma, who received a regimen including brentuximab-vedotin + CHOP before, 3 cycles and 6 cycles after BV-CHOP therapy, respectively. The moderately enhanced FDG uptake on the post-induction (after six cycles of immunochemotherapy) image is due to brown fat activity. There is no clinical or radiological sign of relapse since the last PET/CT scan.

The grey-zone lymphoma diagnosis was a primary diagnosis in 3 of our patients while in another three cases the intermediate form was diagnosed as a result of a revision followed unusual clinical behavior. In the remaining three cases repeated histological sampling was performed during the course of the disease and the examination of the repeat biopsy confirmed an entity intermediate between cHL and DLBCL (one primary refractory case, baseline diagnosis of DLBCL and two relapses: 78 and 35 months after primary treatment, respectively, with cHL as baseline diagnosis). Examination of the immunohistochemical features revealed that 3/9 patients’ samples were CD20 negative whereas CD15 and/or CD30 as well as MUM1 staining were positive in every case. Epstein-Barr virus’s (EBV) association was examined using examination of latent membrane protein (LMP-1), and *in-situ* hybridization of EBER virus sequences, which were negative in each sample. Examination of PD-L1 expression in the HRS cell components revealed strong granular positivity in two-thirds (6/9) of the cases, while samples were negative in one third (3/9) of them**.** Immunohistochemical features are summarized in Table 2. Based on primary histological results, CHOP (cyclophosphamid, adriablastin, vincristin, and prednisolone) protocol was administered in six cases, and ABVD (adriblastin, bleomycin, vinblastin, and dacarbazine) chemotherapy was started in the remaining three patients. In the latter group, therapy was changed to CHOP protocol in one case after only one cycle of ABVD chemotherapy, based on the results of the histological revision. ABVD treatment was finished according to the initial therapeutic plan in two patients, whose baseline histological diagnosis was cHL and it was only the repeat biopsy performed during the relapse of the disease that confirmed intermediate lymphoma. Six patients were given rituximab (anti-CD20 monoclonal antibody) treatment as part of first-line protocol added to CHOP treatment, and in one case CHOP was complemented with brentuximab vedotin (in a compassionate use program, based on the unfavourable prognosis of the histological diagnosis, disseminated disease and CD30 positivity). As a result of the combined therapy of brentuximab-vedotin + CHOP complete metabolic remission was achieved even after three treatment cycles, as shown by the results of a PET/CT scan and was again confirmed by restaging examination (Figure 3). The patient is currently receiving brentuximab-vedotin therapy as maintenance and is in complete remission (14 months have passed since the end of induction therapy). Patients diagnosed with cHL reached complete remission after first-line treatment but the therapeutic response proved to be (more) lasting only in one of the cases (Time to treatment failure: 7 and 39 months). Six patients suffered from primary chemorefractory disease.

**TABLE 2:** Results of the immunohistochemical examination of 9 patients with grey-zone lymphoma ND: no data.

Patient	CD20	CD30	CD15	PAX5	OCT2	CD4	LMP	PD-1L
#1	−	+	−	+	+/−	+/−	−	Granular positive
#2	+	+	−	+	+	+/−	−	Granular positive
#3	+	+	−	ND	+	+/−	−	Granular positive
#4	+	+	ND	+	+/−	+	−	Granular positive
#5	−	+	+/−	+	ND	+	−	Granular positive
#6	−	−	+	ND	+/−	+	−	Negative
#7	Inconclusive	+	−	+	+/−	+	−	Granular positive
#8	+	+	−	+	+	+/−	−	Negative
#9	+	+	−	+	+	−	−	Negative

In the relapsed/refractory cases primarily R-DHAP protocol was started as salvage treatment and, in eligible patients, it was followed by autologous hematopoetic stem cell transplantation performed with R-BEAM conditioning (5/9) patients). We lost only one of the five patients who underwent aHSCT, whose risk of the intervention was already increased due to her comorbidities and poorer performance status. Relapse occurred in one case after autologous HSCT, which was suitable for local irradiation. An elderly female patient, who was not eligible for transplantation, was given a rituximab + gemcitabin regime as salvage therapy while a young refractory male patient received brentuximab-vedotin + bendamustin after the ineffectiveness of salvage DHAP therapy, and then, following another therapeutic failure, was given nivolumab, but he died due to the progression of his disease: he did not live to undergo transplantation.

Our patients’ median overall survival was 60.5 (8–138) months, whereas in the case of our patients who underwent transplantation the corresponding rate was 96.2 (21–138) months.

## Discussion

Gray zone lymphoma, an entity intermediate between diffuse large B-cell, mainly primary mediastinal large B-cell lymphoma and classic Hodgkin lymphoma, especially its nodular sclerosis subtype, was first introduced as a provisional entity in the 2008 WHO classification [2]. No independent, consensual diagnostic criteria have since been defined but, based mainly on the data of retrospective studies, the pathological features of the disease can be summarized as follows. The morphology of tumor cells can be immature centroblastic, and immunoblastic or they can show a Hodgkin-Reed-Sternberg-like transformation. Typically, occurrence and density of tumor cells is greater than in cHL and they often spread in an infiltrative manner. The cumulative number of background cells can be significant, exhibiting mixed composition and not infrequently fibrotic stoma also develops [3–6].

Besides varying degrees of CD30 and/or CD15 positivity, characteristic B-sell markers (CD20, PaX5, CD79a, MUM1, and OCT2) can also be detected with immunostaining, but their expression shows unique combinations. On the whole, it can be concluded that two types of histological patterns can be detected: in one, in addition to the DLBCL-type morphology, B-cell marker expression of tumor B-cells is scarce, while their CD30 or possible CD15 expression is strong. The other pattern is characterized by cHL-type morphology, with weaker or absent CD30/CD15 staining and intensive B-cell marker expression [3–6]. Within the pattern, the histological picture can change zonally; too, sometimes the two types of lymphoma can be spatially distinct, with the morphology suggesting a parallel occurrence of two distinct entities (synchronous lymphoma). Extensive study of histological changes is indispensible for even an experienced hematologist hence core biopsy sampling often has limited value in the diagnosis of GZL, and excision of the complete lymph node is preferred. What may alert the pathologist is the contrasting morphological and immunohistological pictures, which can warrant detailed study, possibly further immunostaining or indicate repeated sampling [6–8]. In our patients the two patterns could be clearly distinguished with initial DLBCL morphology in 6 cases and cHL (morphology) in 3. Primary examination led to GZL diagnosis in three cases while in another three revision was required due to unusual clinical presentation; additional immunostaining led to the precise and final diagnosis of the intermediate histological type.

When gray zone lymphoma was first described, the process was mainly believed to be of mediastinal localization, affecting mostly young, male patients. However, literature reports published in recent years have shown that the non-mediastinal form is just as, if not more common than the cases with primarily mediastinal involvement [3, 4, 7]. There are differences in the features of the two clinical presentations: while patients with MGZ tend to be younger, at earlier stages of the disease and often have bulky tumors, cases with NMGZ lymphoma are characterized by older age, more advanced stages of the disease, extensive extranodal involvement, and frequent bone marrow infiltration. While in MGZL, progression-free survival rates are usually significantly better than among patients with NMGZL, there are no genuine differences between the two groups of patients in terms of survival rates. In only two of our patients did we encounter MGZL, whereas in 7/9 cases we found non-mediastinal advanced disease and female predominance (6/9). The (modest) number of cases does not allow comparison of the two clinical presentations and, perhaps due to the more dominant presence of NMGZL, clinical characteristics also tend to correspond to this subtype: higher age at diagnosis, advanced stage of the disease (7/9 cases), and more common bone marrow infiltrations (5/9 cases) (Table 1).

Due to the rare nature of the entity and the modest number of cases–e.g., in our total number of 680 HL and DLBCL patients only 9 (1.3%) cases proved to be GZL-we have no prognostic systems or clear therapeutic algorithms at our disposal. The disease can be managed using DLBCL-type (R-CHOP, DA-EPOCH-R), or cHL-type (ABVD, escBEACOPP) protocols. Literature data and studies with larger numbers of cases show that DLBCL-type treatments appear to be more effective [3–8]. However, experts’ opinions on the use of rituximab are less clear: while Evens et al. [3] found therapies with additional rituximab more favourable in terms of progression-free survival, Sarkozy et al. [5] did not find rituximab-containing protocols more favourable either for event-free or for overall survival. Comparison of the two studies is made difficult by the fact that, while Evens worked with cases of both MGZL and NMGZL, Sarkozy’s work included only patients with MGZL. Among our own patients CHOP chemotherapy was predominant and ABVD therapy was given only to those patients whose initial histological examination confirmed cHL, and GZL was confirmed only by the repeat biopsy performed during relapse. During their first treatment a total of 6 patients received rituximab; besides the two HL patients treated with ABVD there was one more patient who did not receive rituximab, she was initially diagnosed with GZL and was treated with brentuximab-vedotin in addition to the CHOP regime due to CD20 negativity and strong CD30 positivity. Scarce data are available on the use of brentuximab vedotin; one case has been published where BV was combined with R-CHP [7], and in a phase I/II clinical trial PMBL and DLBCL patients also received the same therapeutic combination (BV + R-CHP) [9]. Brentuximab vedotin can be effective in CD30 positive DLBCL cases [10]. In case of our patient, after complete metabolic remission was achieved with the induction treatment we applied brentuximab vedotin as a maintenance therapy in a similar manner to how it is used in cHL, so far effectively and without significant toxicity. Brentuximab-vedotin therapy can be a logical choice in CD30 positive (but maybe even in negative) cases, but prospective studies are warranted to analyze the precise combination of drugs, their placement (frontline, relapsed), as well as the necessity and advantage of the maintenance therapy.

GZL is characterized by refractoriness to therapy, and often only partial remission is achieved as a result of the first-line treatment followed by early relapse [3–6, 8], a finding that we have experienced with our own patients as well. However, salvage therapy can be successful in relapsed cases [4]. High-dose therapy and hematopoietic stem cell transplantation (HSCT), especially its autologous form, can be an effective therapeutic option in eligible patients and can be one of the pillars of reaching complete remission and, in some cases, of complete cure [3, 4, 6, 8, 11]. Five of our patients received autologous HSCT after R-BEAM conditioning. We lost one patient due to treatment-related complications; her comorbidities increased the risk of the intervention. Following HSCT, one patient developed cervical lymph node relapse after more than two years of remission, which was suitable for radiotherapy due to its localized nature. One young patient did not live to undergo transplantation; he died during progression of his disease despite several salvage treatments (DHAP, BV-bendamustin, and nivolumab). The place of PD-1 inhibitor treatment is still unclear as is its role in the management of GZL, but, according to the literature, it has been successfully used in relapsed/refractory cases [12]. The relevance of using PD-1 inhibitors is supported by the fact that in 60% of MGZL cases copy number variation in the PD-1 gene located in the 9p24.1 chromosomal region could be detected. In six out of nine of our cases presented here we were able to confirm PD-L1 positivity.

There are also ongoing clinical trials with polatuzumab-vedotin and blinatumomab in various types of B-cell lymphomas and both of them can have a potential beneficial effect in the treatment of gray zone lymphoma also.

Managing GZL is challenging from diagnosis to treatment of the patient across the whole spectrum. It is of utmost importance that, unusual histological presentation or clinical behaviors should raise physicians’ suspicion and clinicopathological consultation is warranted about the case and, if necessary, the diagnosis and/or treatment should be reconsidered. In therapy, a more aggressive approach and DLBCL-type protocols can be more effective; while the therapeutic impact of rituximab is unclear, it can be useful in CD20 positive cases. The role of brentuximab-vedotin is likewise unclear and we have significantly less experience with it. However, its use should be considered even as a front-line therapy in CD30 positive cases, while in relapsed or refractory patients its benefit seems to be clearer. In the case of patients with a weaker general condition maintenance BV therapy can help in turning partial into complete remission or, like in cHL, in deepening therapeutic response after autologous transplantation. In refractory/relapsed cases, the role of transplantation, primarily that of autologous HSCT is unquestionable but the issue is in which cases it can be considered for use as a frontline therapy [8]. So far, PD-1 inhibitors have been mostly used in relapsed/refractory cases but in older, more fragile patients they can even represent an alternative to transplantation. In conclusion, there are still numerous open questions in the management of GZL that are difficult to answer due to the rare nature of the disease. Prospective, multicentered clinical studies are warranted to gain more information about the disease’s pathobiology and clinical characteristics and, based on the results, develop more effective treatment strategies.

## Data Availability

The raw data supporting the conclusions of this article will be made available by the authors, without undue reservation.
